# Foreword to the special virtual issue on *Modern approaches and tools for teaching crystallography*

**DOI:** 10.1107/S2056989023006667

**Published:** 2023-08-01

**Authors:** Graciela Díaz de Delgado, Sean Parkin

**Affiliations:** a Laboratorio de Cristalografía-LNDRX, Departamento de Química, Facultad de Ciencias, Universidad de Los Andes Mérida, Venezuela; b Department of Chemistry, CP-110, Chemistry-Physics Building, University of Kentucky, Lexington, KY 40506-0055, USA

**Keywords:** crystallographic teaching, space-group symmetry, crystal growing, twinning, validation criteria, disorder, electron diffraction

## Abstract

A compilation of articles with a strong teaching element published since 2018 is presented alongside an overview of the articles in the special issue on this topic.

Since its early days, crystallography has proven to be a highly interdisciplinary and collaborative science. At the same time, it stands out as a field whose members are strongly committed to education. Crystallographers are well recognized for their dedication to teaching. The determination of the structure of even a simple molecule could become complicated by disorder, twinning, incommensurate modulation, low crystal quality, *etc*. In addition, analysis of geometrical parameters and intermolecular interactions, graphical representation, structure validation, and manuscript publication often require the help of an expert crystallographer to take the results to publication in a prestigious journal.

At the core of IUCr activities is the sponsorship of schools and workshops around the world on different aspects of crystallography, from basic and advanced concepts in symmetry, to structure determination of small and large molecules, powder and single-crystal diffraction techniques, to new experimental methods, among other topics. Many young crystallographers have benefited from attending prestigious crystallography schools and workshops such as the International School of Crystallography in Erice, the ACA Summer Course on Crystallography, the European Crystallography School, *etc*. These efforts, strongly supported by the IUCr, aim to compensate for the absence of formal courses in crystallography at the undergraduate and graduate degree levels, as noted by Kantardjieff, Kaysser-Pyzalla, and Spadon in the foreword of the special issue ‘Crystallography education and training for the 21st century’ in the *Journal of Applied Crystallography* (Kantardjieff *et al.*, 2010 ▸).

It is well recognized that the crystallographic community rapidly adapts to new technological advances, in many cases even driving them. This community is also always looking for new approaches and teaching tools, and makes extensive use of online resources, as became evident during the lockdown measures established globally to control the spread of Covid-19. As soon as it was possible, teaching, research, collab­oration, meetings, schools, and workshops resumed, using different meeting platforms. Since online teaching requires a great deal of preparation, many crystallographers worked hard to develop online content for students and newcomers to the field, some produced step-by-step guides to the solution of difficult problems, and others dedicated time to contribute to the teaching of fundamental concepts.

The special issue (https://journals.iucr.org/special_issues/2023/teaching/index.html) features contributions by B. M. Foxman (Foxman, 2021 ▸) and by S.-L. Zheng and M. G. Campbell (Zheng & Campbell, 2021 ▸) on teaching space groups. Foxman’s approach consists of a tutorial, conceived and written with the late Jerry Jasinski, that presents the crystal classes in five modules of more than 200 PowerPoint ‘slides’. Humor (a distinctive trademark of Professor Foxman), historical context, hypothetical dialogues between ‘Great Figures of Crystallography’, and Q&A combine into an entertaining pedagogical presentation of space-group symmetry.

Zheng & Campbell (2021 ▸) then describe how they use Foxman’s tutorial in a ‘peer-tutoring’ approach to teaching space-group diagrams. Through explaining to their peers the recently acquired understanding of symmetry and space-group diagrams, students are able to consolidate the concepts learned and become more engaged in learning crystallography.

The importance of a good understanding of space-group symmetry is evident in the paper by van Terwingen & Englert (2022 ▸). The structure of tri­methyl­pyrazole, a very simple heterocycle, which crystallizes in the interesting space group *Pnma* is reported. The authors describe the structure in terms of Kitaigorodskii’s and Wilson’s ideas of crystal packing.

Bart Kahr (Kahr, 2023 ▸) presents us with a very interesting idea: the design of a periodic-like table of space groups. The table is constructed based on the symmorphic space groups arranged over two non-linear non-orthogonal axes. The corresponding non-symmorphic space groups are treated as ‘isotopes’. As Kahr suggests, such a table would be of value to expert and novice crystallographers as well as researchers in solid-state sciences. The design is certainly open to improvements or to new design ideas.

Crystal-growth experiments provide excellent opportunities to discuss many fundamental concepts such as crystal packing, use of crystallographic databases, determination of physicochemical properties, stereoisomerism, among many others. Crystal-growing competitions have become one of the most important and engaging outreach activities of IUCr. Wouters & Van Meervelt (2022 ▸) present a simple procedure to grow large crystals of the artificial sweeteners erythritol and xylitol coupled with the determination of the heat of dissolution using a simple calorimetric set-up.

Four papers in the issue contain detailed advice on dealing with complications during structure solution and refinement. Parkin (2021 ▸) uses three case studies to provide hints and tips to deal with pseudo-merohedric twins. Milewski *et al.* (2022 ▸) alert us to the need to always examine critically the results of an automated structure determination. Vinaya *et al.* (2023 ▸) present a step-by-step guide to modeling whole-molecule disorder. Parkin, Cunningham *et al.* (2023 ▸) discuss in detail validation criteria for the structure determination of a mixed phosphine sulfide/selenide. These contributions contain excellent examples to use in schools and workshops, complemented by other articles across IUCr Journals dealing with, for example, pitfalls in the automatic determination of symmetry (Clegg, 2019 ▸), complex cases of disorder (Parkin, Glidewell *et al.*, 2023 ▸), validation (Linden, 2020 ▸; Spek, 2020 ▸), treatment of hydrogen atoms (Fábry, 2018 ▸), Hirshfeld surface analysis (Tan *et al.*, 2019 ▸), to name just a few.

Simoncic *et al.* (2023 ▸) give us an overview of the advances, applications and challenges of electron diffraction, a technique that will surely become of importance for the characterization of crystals less than 1 µm in size of pharmaceuticals and other materials with potential technological applications.

To conclude the issue, Massera & Helliwell (2023 ▸) share their list of ‘Golden Oldies’. The authors suggest ten crystallography articles that must be read, reformulating the title of each paper to emphasize the importance of that concept and how it is perceived and applied today. With about 500 downloads on the day it was published (June 6, 2023) and more than 5100 downloads up to July 28, this article is attracting a great deal of attention and shows the appreciation that crystallographers have for the history of our field and their commitment to teaching.

Routine structure determination and refinement from powder and MicroED data, the implementation of artificial intelligence (AI) and machine learning (ML) tools, the availability of more powerful radiation sources, among many other advances in our field, will certainly require a sustained teaching effort. We considered that a special issue on teaching in *Acta Crystallographica Section E* was long overdue. We hope this will be the seed of a permanent section dedicated to teaching, not only in *Acta E* but also across all IUCr journals. We look forward to further contributions.
